# Risk factors of extra-hepatic progression after transarterial chemoembolization for hepatocellular carcinoma patients: a retrospective study in 654 cases

**DOI:** 10.7150/jca.35355

**Published:** 2019-08-27

**Authors:** Shaohua Li, Qiaoxuan Wang, Jie Mei, Jianwei Wang, Xiao-Ping Zhong, Yihong Ling, Zhixing Guo, Liang-He Lu, Wei Wei, Rongping Guo

**Affiliations:** 1 Department of Hepatobiliary Oncology of the Sun Yat-sen University Cancer Center; Guangzhou 510060, P.R. China; 2 Department of Radiation Oncology of the Sun Yat-sen University Cancer Center, Guangzhou 510060, P.R. China; 3 Department of Ultrasound of the Sun Yat-sen University Cancer Center, Guangzhou 510060, P.R. China; 4 Department of pathology of the Sun Yat-sen University Cancer Center, Guangzhou 510060, P.R. China; 5 State Key Laboratory of Oncology in South China, Guangzhou 510060, P.R. China; 6 Collaborative Innovation Center for Cancer Medicine, Guangzhou 510060, P.R. China; 7 Department of Burn and Plastic Surgery, 2nd Affiliated Hospital of Shantou University Medical College, Shantou 515041, China

**Keywords:** hepatocellular carcinoma, transarterial chemoembolization, extrahepatic progression, risk factor

## Abstract

**Aim**: To investigate the risk factors of extra-hepatic progression after TACE in HCC.

**Methods**: The study population included 654 HCC patients who underwent TACE between October 2005 and September 2012. We collected and analyzed their clinical characteristics and survival information. TACE was performed as previously described with minor modifications. When necessary, superselective chemoembolization was performed through the segmental or subsegmental arteries, based on the tumor location and extent and hepatic function reserve. If stasis could not be achieved in a tumor-feeding artery, iodized oil was used solely in some patients. Embolization was then performed with injection of absorbable gelfoam particles (1-2 mm in diameter) through the angiographic catheter.

**Results**: The tumor response to initial TACE was evaluated in 645 patients. The CR rate, response rate (RR), and disease control rate (DCR) were 9.92%, 25.89%, and 70.39%, respectively. The median overall survival (OS) period was 14.5 months. The 6-month, 1-, 2-, 3-, and 5-year OS rates were 75.5%, 55.0%, 33.9%, 22.8%, and 14.9%, respectively. The median progression-free survival (PFS) period was 4.3 months. The 6-month, 1-, 2-, 3-, and 5-year PFS rates were 40.7%, 27.1%, 16.7%, 13.9%, and 9.3%, respectively. One hundred and fifty patients developed extrahepatic progression during follow-up. We demonstrated that in the absence of radical treatment after initial TACE (p<0.001), the presence of extrahepatic metastasis before initial TACE (p<0.001), AST >45 U/L (p=0.024), ALB <35 g/L (p=0.012), and tumor response were evaluated as PD and SD after initial TACE (p<0.001) and were found to be independent predictors of a poorer prognosis of extrahepatic PFS.

**Conclusions**: We identified risk factors for extrahepatic progression after TACE in HCC patients. Early combination treatment was strongly recommended in patients that met these risk factors.

## Introduction

Hepatocellular carcinoma (HCC) is one of the most commonly diagnosed cancers and causes of cancer mortality worldwide.^1^, ^2^ Fewer than 20% of HCC patients can receive curative therapies such as surgical resection, liver transplantation and ablative therapies due to advanced disease at presentation or poor liver functional reserve.^3^ Transarterial chemoembolization (TACE) is a widely accepted treatment for unresectable or intermediate-stage HCCs.^4^-^6^ However, their prognosis remains poor.^6^, ^7^ Extrahepatic progression following TACE is one of the important factors. However, very few studies have been reported to evaluate the risk factors for extrahepatic progression after TACE in HCC patients.

This retrospective study was designed to investigate the risk factors for extrahepatic progression after TACE in HCC patients in order to improve individualized treatment and promote precision treatment of these patients.

## Materials and Methods

This research was approved by the institutional review board (IRB) of Sun Yat-sen University Cancer Center. The approval number is B2018-134-01. The experiments were carried out in accordance with the approved guidelines.

### Patients

Between October 2005 and September 2012, 1635 patients were newly diagnosed with HCC and received TACE as primary treatment in our department. Of these, 654 (40%) cases with complete medical records were included into the current retrospective study.

The diagnosis of HCC was based on the diagnostic criteria for HCC described in the American Association for the Study of the Liver (AASLD) guidelines.^5^ Routine pretreatment examination included blood chemistry, serum tumor biomarker such as alpha-fetoprotein (AFP), chest radiography, ultrasonography, tri-phase contrast-enhanced helical computed tomography (CT), and/or contrast-enhanced magnetic resonance (MR) imaging of the abdomen. Further investigations were performed whenever there was clinical suspicion of extrahepatic metastases. Liver function was evaluated based on the Child-Pugh classification system 8 and the indocyanine green (ICG) clearance test was performed routinely within three days before treatment. The selection criteria for the TACE procedure depended on the tumor location and extent, the liver function and the general condition of the patient.

### TACE procedure

TACE was performed as previously described with minor modifications.^6^ When necessary, superselective chemoembolization was performed through the segmental or subsegmental arteries, based on the tumor location and extent and hepatic function reserve. If stasis could not be achieved in a tumor-feeding artery, iodized oil was used solely in some patients. Embolization was then performed with injection of absorbable gelfoam particles (1-2 mm in diameter) through the angiographic catheter.

### Post-TACE care and follow-up

The posttreatment care and follow-up were performed routinely in all patients. A serum AFP assay, liver function test, and abdominal ultrasonography or tri-phase contrast-enhanced helical CT were performed monthly during the first three months. Thereafter, the patients were followed every 2-3 months with radiology and serum examination. Further investigations were performed as needed.

The modified Response Evaluation Criteria in Solid Tumors (mRECIST) was used to evaluate tumor response 9. The first evaluation was performed one month after TACE. Another TACE treatment was performed every 4-8 weeks until one of the following end points was reached: (1) complete devascularization of the tumor, (2) technical impossibility to embolize the residual tumors, (3) development of contraindications to embolization, and (4) total resection or ablation of the tumor by subsequent surgery or local ablation. The proper subsequent treatment was defined as the clinical routine. The follow-up ended on January 31, 2018.

### Statistical analysis

Comparisons between categorical variables were performed using Pearson's χ2 test or Fisher's exact test where appropriate. Continuous variables were compared using Student's t-test (when values were normally distributed) or the Mann-Whitney test (when values had a distribution that departed significantly from normal). The survival analysis was calculated using the Kaplan-Meier method and compared using the log-rank test. Univariate and multivariate analyses using Cox' proportional hazard model were performed to evaluate the prognostic factors. The correlation between two variables was examined by Pearson's correlation analysis (when values were normally distributed) or Spearman's correlation analysis (when values had a distribution that departed significantly from normal). A value of p<0.05 was considered statistically significant. All data were analyzed using SPSS statistical software for Windows (ver. 18.0; SPSS Inc., Chicago, IL, USA).

All continuous variable data are expressed as the mean ± standard error (when values were normally distributed) or the median (range) (when values had a distribution that departed significantly from normal). All data regarding categorical variables are shown as n (proportion).

## Results

### Baseline clinical characteristics

The baseline clinical characteristics of 654 HCC patients treated with TACE are summarized in Table 1. In the study cohort, the mean age was 51.23 years; most of the patients were male (603 cases, 92.20%) and hepatitis B surface antigen positive (576 cases, 88.07%). Serum AFP level was higher than 400 ng/ml in approximately half of the patients (332 cases, 50.76%). The liver function was good in most of the patients classified as Child-Pugh A (564 cases, 86.24%) and B (85 cases, 13.00%); the median ICG 15 minute retention (ICGR15) was 6.1%. There were 249 cases (38.07%) staged as stage A, 160 cases (24.46%) as stage B, 243 cases (37.16%) as stage C, and 2 cases (0.31%) as stage D, according to the BCLC algorithm. Seventy cases (10.70%) had extrahepatic metastases, and 207 cases (31.65%) had portal/hepatic vein tumor thrombosis.

### Tumor response and subsequent treatment

The tumor response to initial TACE was evaluated in 645 patients, as shown in Table 1. The CR rate, response rate (RR), and disease control rate (DCR) were 9.92%, 25.89%, and 70.39%, respectively.

The subsequent treatment was chosen based on the tumor location and extent, the liver function and the general condition of the patient as shown in Table 2. Overall, 172 cases (26.30%) received 342 cycles of radical treatments afterwards.

### Survival and progression

**Figure 1:**
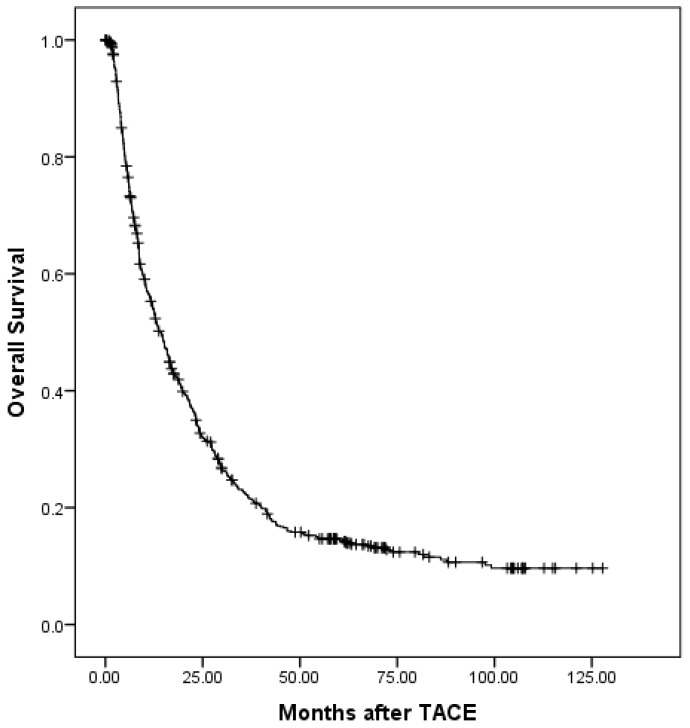
The overall survival of all patients.

**Figure 2:**
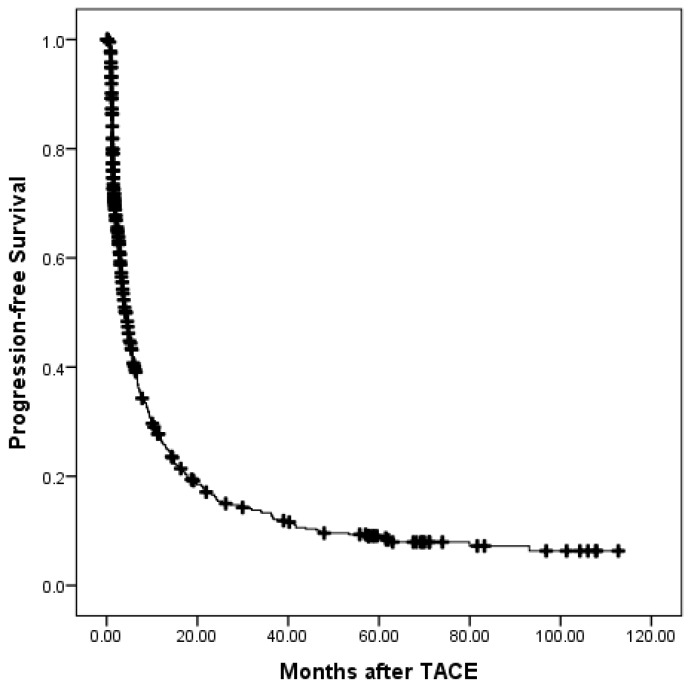
The progression-free survival of all patients.

At a median follow-up time of 12.7 months (range, 0.1-127), 532 patients (81.35%) had died. Of these, 4 patients died without any radiographic progression, which were counted in extrahepatic progression as shown in Table 3. The median overall survival (OS) period was 14.5 (95% confidence interval (CI), 12.6-16.3) months. The overall survival is shown in Fig. 1. The 6-month, 1-, 2-, 3-, and 5-year OS rates were 75.5%, 55.0%, 33.9%, 22.8%, and 14.9%, respectively. During the follow-up period, 488 patients (74.62%) had disease progression. The median progression-free survival (PFS) period was 4.3 (95% CI, 3.6-5.0) months. The progression-free survival is shown in Fig. 2. The 6-month, 1-, 2-, 3-, and 5-year PFS rates were 40.7%, 27.1%, 16.7%, 13.9%, and 9.3%, respectively. Overall, 150 patients developed extrahepatic progression as shown in Table 3.

### Prognostic factors for overall survival

Univariate and multivariate analysis of factors affecting OS are shown in Table 4. Age ≤50 yr, having not received radical treatment after initial TACE, a maximum tumor diameter≥10 cm, the presence of extrahepatic metastasis before initial TACE, the presence of portal/hepatic vein tumor thrombosis before initial TACE, AFP >400 ng/ml, platelet count (PLT)>100×109/L, alanine aminotransferase (ALT) >40 U/L, aspartate transaminase (AST)>45 U/L, albumin (ALB) <35 g/L, total bilirubin (TBil) >20.5 μmol/L, Child-Pugh score >6, B/C/D stage according to the BCLC staging system, lipiodol dose during TACE >15 ml, the use of gelfoam during TACE, and tumor response that were evaluated as PD and SD after initial TACE and found to be statistically significant by univariate analysis were included in a multivariate regression analysis. The results of the latter demonstrated that not having received radical treatment after initial TACE (p<0.001, HR: 2.868; 95% CI: 2.292-3.589), a maximum tumor diameter ≥10 cm (p=0.016, HR: 1.327; 95% CI: 1.054-1.669), the presence of portal/hepatic vein tumor thrombosis (p=0.004, HR: 1.427; 95% CI: 1.120-1.817), AFP >400 ng/ml (p=0.001, HR: 1.353; 95% CI: 1.125-1.629), AST >45 U/L (p=0.001, HR: 1.534; 95% CI: 1.200-1.960), and a tumor response that had been evaluated as PD and SD after initial TACE (p<0.001, HR: 1.829; 95% CI: 1.480-2.261) were independent predictors of poorer prognosis of OS.

### Prognostic factors for progression-free survival

Similarly, univariate and multivariate analyses of factors affecting PFS are shown in Table 5. Not having received radical treatment after initial TACE, a maximum tumor diameter ≥10 cm, the presence of extrahepatic metastasis before initial TACE, the presence of portal/hepatic vein tumor thrombosis before initial TACE, AFP >400 ng/ml, white blood cell count (WBC) >4.0×109/L, PLT >100×109/L, AST >45 U/L, ALB <35 g/L, Child-Pugh score >6, stage B/C/D according to the BCLC staging system, lipiodol dose during TACE >15 ml, and tumor response that were evaluated as PD and SD after initial TACE and found to be statistically significant by univariate analysis were included in a multivariate regression analysis. The results of the latter revealed that not having received radical treatment after initial TACE (p<0.001, HR: 1.663; 95% CI: 1.347-2.053), portal/hepatic vein tumor thrombosis (p=0.021, HR: 1.339; 95% CI: 1.044-1.717), AST >45 U/L (p=0.024, HR: 1.279; 95% CI: 1.032-1.586), and tumor response that were evaluated as PD and SD after initial TACE (p<0.001, HR: 2.032; 95% CI: 1.629-2.534) were independent predictors of poorer prognosis of PFS.

### Prognostic factors for extrahepatic progression-free survival

Additionally, univariate and multivariate analyses of factors affecting extrahepatic PFS are shown in Table 6. Age ≤50 yr, not having received radical treatment after initial TACE, maximum tumor diameter ≥10 cm, the presence of extrahepatic metastasis before initial TACE, the presence of portal/hepatic vein tumor thrombosis before initial TACE, AFP >400 ng/ml, PLT >100×109/L, AST >45 U/L, ALB <35 g/L, stage B/C/D according to the BCLC staging system, lipiodol dose during TACE >15 ml, and tumor response that were evaluated as PD and SD after initial TACE and found to be statistically significant by univariate analysis were included in a multivariate regression analysis. The results of the latter revealed that not having received radical treatment after initial TACE (p<0.001, HR: 2.629; 95% CI: 1.697-4.072), the presence of extrahepatic metastasis before initial TACE (p<0.001, HR: 2.259; 95% CI: 1.432-3.563), AST >45 U/L (p=0.024, HR: 1.612; 95% CI: 1.065-2.439), ALB <35 g/L (p=0.012, HR: 1.803; 95% CI: 1.135-2.862), and tumor response that were evaluated as PD and SD after initial TACE (p<0.001, HR: 2.608; 95% CI: 1.670-4.071) were independent predictors of poorer prognosis of extrahepatic PFS.

## Discussion

Although TACE has been widely used as a palliative therapy worldwide, especially in China and other Asian countries 6, 10-12, single TACE treatment is not recommended whenever extrahepatic metastasis is present 5, 13. The BCLC staging classification recommends the administration of sorafenib as the first line treatment for HCC patients who have extrahepatic metastasis 5, 14. A previous study showed that the prognosis of HCC patients with extrahepatic metastasis is significantly worse than that of advanced HCC patients without extrahepatic metastasis 15. These results suggest that advanced HCC patients with extrahepatic metastasis or extrahepatic progression after TACE need combination treatments with TACE and systematic therapy, including sorafenib or radiotherapy 4, which makes identifying the risk factors for extrahepatic progression after TACE a matter of considerable importance.

In the present study, several factors were found to be associated with OS and PFS. Some factors are well accepted in previous reports, such as tumor size, portal vein invasion, and AFP level 16-18. Of note, while the tumor size, portal vein invasion, and AFP level were associated with OS and PFS, these factors were not significant predictors of extrahepatic progression in the present study, suggesting that in advanced HCC, the tumor burden in the liver itself is unrelated to extrahepatic progression after TACE treatment. It is controversial from the clinical point of view and numerous previous studies. In univariate analysis these factors are of significance. However, these may be obscured by other factors in the multivariate analysis. Although AFP can show good prognostic ability in most of the time, it is not an absolutely accurate prognostic indicator for extrahepatic progression. Tumor thrombi are meaningful in both multivariate analysis of OS and PFS, but show no significance in extrahepatic PFS. It may indicate that tumor thrombus is more likely to cause intrahepatic dissemination, rather than the progress of extrahepatic lesions in TACE treatment.

In the present study, the presence of extrahepatic metastasis did not affect OS and PFS, which is inconsistent with a previous study 15. However, the presence of extrahepatic metastasis before TACE is an independent risk factor for extrahepatic progression. The results may be ascribed to the patients with extrahepatic metastasis suitable to accept combination treatment strategy such as systematic treatment and/or radiotherapy, which could enhance the anti-tumor effect compared to TACE alone.

Similarly, hypoproteinemia (albumin lower than 35 g/L) is a unique risk factor for extrahepatic progression, while other biomarkers for liver function such as total bilirubin, prothrombin time, ICGR15, alanine aminotransferase, and aspartate aminotransferase did not demonstrate prognostic prediction power for extrahepatic progression. This observation may suggest that albumin level plays quite a unique role in HCC patients, especially advanced patients. The treatment options of a significant number of patients may be limited by hypoproteinemia, making repeat TACE, combination therapy or other treatments impossible.

An increased AFP level has been associated with larger tumors and lower hypohepatia, reflecting an aggressive biology 19. In present study, AFP >400 ng/ml is associated with OS, which is consistent with a previous report 20. However, no association was found with PFS and extrahepatic PFS, suggesting that TACE may inhibit the aggressive behavior of high AFP level tumors; this hypothesis needs further verification.

Of note, in the present study, extrahepatic metastasis present before TACE is an independent risk factors for extrahepatic progression after TACE, which is consistent with previous report 21. Leal, et al. mentioned the patterns of progression were different between patients with and without extrahepatic metastasis. Based on the results of present and previous studies, we suggest apply combination therapy including target therapy and/or radiotherapy as early as possible in patients with extrahepatic metastasis.

We acknowledge some weaknesses in our study. First, the nature of retrospective study brought choose bias inevitably, the results needs further prospective study to confirm. Second, the chemotherapy regimen in TACE were various, which made the analysis more complex while making the results closer to real clinical practice at the same time in fact. Finally, to obtain the generalizability of our results, another validation cohort from other centers rather than our single center might be necessary.

In conclusion, we identified that the presence of extrahepatic metastasis before TACE, AST >45 U/L, ALB <35 g/L, and lack of response after TACE as independent risk factors for extrahepatic progression. To gain better therapeutic outcome and survival, early combination treatment including target therapy and/or radiotherapy was strongly recommended in these patients.

## Figures and Tables

**Table 1:** Baseline clinical characteristics and evaluation of tumor response after initial TACE in 654 HCC patients

Baseline clinical characteristics	n=654; mean±SE, median (range), or n (proportion)
Age (years)	51.23±0.48
Gender	
Male	603 (92.20%)
Female	51 (7.80%)
HBsAg status	
Negative	65 (9.94%)
Positive	576 (88.07%)
Unknown	13 (1.99%)
Preoperative AFP level	
≤400 ng/ml	322 (49.24%)
>400 ng/ml	332 (50.76%)
Preoperative ALT level (U/L)	45.4 (6~667.4)
Preoperative AST level (U/L)	57 (13~521)
Preoperative TBil level (umol/L)	15.4 (2.92~128)
Preoperative ALB level (g/L)	40.15±0.18
Preoperative WBC level (10^9^/L)	6.6 (2~27)
Preoperative PLT level (10^9^/L)	176 (16~594)
Preoperative PT level (s)	12.6 (8.7~22.8)
Preoperative Child-Pugh score	
A5	333 (50.92%)
A6	231 (35.32%)
B7	63 (9.63%)
B8	18 (2.75%)
B9	4 (0.61%)
C10	2 (0.31%)
Unknown	3 (0.46%)
Preoperative ICGR15 (%)	6.1 (0~57.4)
Number of tumors	
Solitary	399 (61.01%)
Multiple	255 (38.99%)
Maximum diameter of tumor (cm)	8.8 (1~22)
Extrahepatic metastasis	
Negative	584 (89.30%)
Positive	70 (10.70%)
Portal/Hepatic vein tumor thrombosis	
Negative	447 (68.35%)
Positive	207 (31.65%)
BCLC stage	
Stage A	249 (38.07%)
Stage B	160 (24.46%)
Stage C	243 (37.16%)
Stage D	2 (0.31%)
Lipiodol dose (ml)	15 (0~70)
Use of gelfoam	
No	543 (83.03%)
Yes	111 (16.97%)
Tumor response to initial TACE (n=645)	
CR	64 (9.92%)
PR	103 (15.97%)
SD	287 (44.50%)
PD	191 (29.61%)
Postoperative hospital stay (days)	5 (1~105)

TACE: transcatheter arterial chemoembolization; HBsAg: hepatitis B surface antigen; AFP: alpha fetoprotein; ALT: alanine aminotransferase; AST: aspartate aminotransferase; TBIL: total bilirubin; ALB: albumin; WBC: white blood cell; PLT: blood platelet; PT: prothrombin time; BCLC: Barcelona Clinic Liver Cancer; CR: complete response; PR: partial response; SD: stable disease; PD: progression disease

**Table 2:** The subsequent treatment following initial TACE of all patients

Treatment	Cases	Cycles
Radical treatment		
Resection for primary lesion	46	49
RFA for primary lesion	85	121
PMCT for primary lesion	64	115
PEI for primary lesion	31	46
Cryoablation for primary lesion	6	8
Transplantation	3	3
Palliative treatment		
TACE	323	555
TAI	9	10
Systemic chemotherapy	19	55
Sorafenib	45	-
Radiotherapy	18	19
Sealed source radiotherapy	7	13
Resection for metastatic lesion	1	1
PMCT for metastatic lesion	4	5
RFA for metastatic lesion	3	6
Cryoablation for metastatic lesion	1	1
CIK cell therapy	5	12

RFA: radiofrequency ablation; PMCT: percutaneous microwave tumor coagulation therapy; PEI: percutaneous ethanol injection therapy; TACE: transcatheter arterial chemoembolization; TAI: transarterial infusion chemotherapy; CIK: cytokine-induced killer

**Table 3:** The characteristics of 150 patients with extrahepatic progression

Site of extrahepatic progression	Cases
Lung	83
Lymph node(s)	24
Bone	13
Lung+lymph node(s)	8
Adrenal gland(s)	7
Death	4
Bone+lymph node(s)	2
Lung+bone	1
Lung+adrenal gland+lymph nodes	1
Peritoneum	1
Chest wall	1
Lung+adrenal gland+chest wall	1
Peritoneum+bone+muscle	1
Lung+peritoneum+pleura	1
Peritoneum+lymph nodes	1
Adrenal gland+lymph nodes	1

**Table 4:** Univariate and multivariate analyses of factors affecting OS

	Univariate Cox regression analysis for OS			Multivariate Cox regression analysis for OS		
Variables	HR	95% CI	p value	HR	95% CI	p value
Male	1.076	0.77-1.503	0.668			
Age (≤50 yr)	1.261	1.063-1.496	0.008	1.071	0.888-1.293	0.472
No radical treatment	3.146	2.54-3.895	0.000	2.868	2.292-3.589	0.000
Single lesion	1.045	0.877-1.245	0.623			
Maximum tumor diameter (≥10 cm)	2.080	1.743-2.483	0.000	1.327	1.054-1.669	0.016
Extrahepatic metastasis present	1.361	1.046-1.772	0.022	0.945	0.707-1.263	0.702
Portal/hepatic vein tumor thrombosis present	2.323	1.933-2.791	0.000	1.427	1.120-1.817	0.004
AFP (>400 ng/ml)	1.677	1.412-1.992	0.000	1.353	1.125-1.629	0.001
WBC (>4.0×10^9^/L)	1.202	0.891-1.623	0.228		-	
PLT (>100×10^9^/L)	1.281	1.022-1.607	0.032	1.090	0.841-1.413	0.513
ALT (>40 U/L)	1.315	1.104-1.567	0.002	0.956	0.77-1.186	0.682
AST (>45 U/L)	1.981	1.645-2.387	0.000	1.534	1.200-1.960	0.001
ALB (<35 g/L)	1.338	1.043-1.717	0.022	1.203	0.886-1.633	0.237
TBil (>20.5 μmol/L)	1.236	1.018-1.502	0.033	1.022	0.813-1.284	0.853
HBsAg positive	1.154	0.868-1.534	0.323		-	
PT (>13.5 s)	1.142	0.942-1.384	0.176		-	
ICGR15 (>10%)	1.166	0.952-1.428	0.138		-	
Child-Pugh score (>6)	1.394	1.08-1.799	0.011	1.240	0.88-1.747	0.219
BCLC stage (B/C/D)	1.436	1.202-1.717	0.000	1.163	0.927-1.459	0.191
Lipiodol dose (>15 ml)	1.684	1.416-2.001	0.000	0.953	0.772-1.177	0.653
Use of gelfoam	1.268	1.017-1.581	0.035	1.054	0.822-1.351	0.680
PD+SD after initial TACE	2.058	1.674-2.53	0.000	1.829	1.480-2.261	0.000

OS: overall survival; AFP: alpha fetoprotein; WBC: white blood cell; PLT: blood platelet; ALT: alanine aminotransferase; AST: aspartate aminotransferase; TBIL: total bilirubin; ALB: albumin; HBsAg: hepatitis B surface antigen; PT: prothrombin time; ICG: indocyanine green; BCLC: Barcelona Clinic Liver Cancer; SD: stable disease

**Table 5:** Univariate and multivariate analyses of factors affecting PFS

	Univariate Cox regression analysis for PFS				Multivariate Cox regression analysis for PFS		
Variables	HR	95% CI	p value		HR	95% CI	p value
Male	1.279	0.902-1.814	0.152				
Age (≤50 yr)	1.151	0.963-1.375	0.122				
No radical treatment	1.883	1.538-2.305	0.000		1.663	1.347-2.053	0.000
Multiple lesions	1.015	0.847-1.217	0.870				
Maximum tumor diameter (≥10 cm)	1.507	1.25-1.818	0.000		1.022	0.813-1.284	0.855
Extrahepatic metastasis present	1.501	1.125-2.004	0.006		1.275	0.934-1.741	0.126
Portal/hepatic vein tumor thrombosis present	1.872	1.543-2.27	0.000		1.339	1.044-1.717	0.021
AFP (>400 ng/ml)	1.280	1.07-1.53	0.007		1.081	0.893-1.308	0.425
WBC (>4.0×10^9^/L)	1.543	1.107-2.149	0.010		1.044	0.718-1.52	0.821
PLT (>100×10^9^/L)	1.335	1.052-1.693	0.017		1.217	0.917-1.615	0.174
ALT (>40 U/L)	1.146	0.956-1.375	0.141				
AST (>45 U/L)	1.503	1.244-1.815	0.000		1.279	1.032-1.586	0.024
ALB (<35 g/L)	1.440	1.105-1.875	0.007		1.260	0.918-1.73	0.152
TBil (>20.5 umol/L)	1.019	0.828-1.254	0.861				
HBsAg negative	1.007	0.753-1.347	0.964				
PT (>13.5 s)	1.071	0.877-1.308	0.501				
ICGR15 (>10%)	1.206	0.978-1.487	0.080				
Child-Pugh score (>6)	1.330	1.015-1.741	0.038		1.292	0.924-1.806	0.134
BCLC stage (B/C/D)	1.370	1.139-1.647	0.001		1.115	0.888-1.401	0.347
Lipiodol dose (>15 ml)	1.341	1.118-1.607	0.002		0.987	0.795-1.226	0.907
Use of gelfoam	1.150	0.914-1.445	0.233				
PD+SD after initial TACE	2.202	1.78-2.725	0.000		2.032	1.629-2.534	0.000

PFS: progression-free survival; AFP: alpha fetoprotein; WBC: white blood cell; PLT: blood platelet; ALT: alanine aminotransferase; AST: aspartate aminotransferase; TBIL: total bilirubin; ALB: albumin; HBsAg: hepatitis B surface antigen; PT: prothrombin time; ICG: indocyanine green; BCLC: Barcelona Clinic Liver Cancer; SD: stable disease

**Table 6:** Univariate and multivariate analyses of factors affecting extrahepatic PFS

	Univariate Cox regression analysis for extrahepatic progression-free survival				Multivariate Cox regression analysis for extrahepatic progression-free survival		
Variables	HR	95% CI	p value		HR	95% CI	p value
Male	1.184	0.64-2.189	0.591				
Age (≤50 yr)	1.723	1.245-2.385	0.001		1.387	0.965-1.994	0.077
No radical treatment	3.093	2.027-4.72	0.000		2.629	1.697-4.072	0.000
Single lesion	1.388	0.985-1.957	0.061				
Maximum tumor diameter (≥10 cm)	2.419	1.743-3.358	0.000		1.143	0.76-1.719	0.520
Extrahepatic metastasis present	2.916	1.939-4.385	0.000		2.259	1.432-3.563	0.000
Portal/hepatic vein tumor thrombosis present	2.577	1.85-3.589	0.000		1.460	0.943-2.261	0.090
AFP (>400 ng/ml)	1.770	1.276-2.454	0.001		1.206	0.842-1.729	0.307
WBC (>4.0×10^9^/L)	1.477	0.819-2.667	0.195				
PLT (>100×10^9^/L)	1.808	1.117-2.927	0.016		1.292	0.755-2.211	0.350
ALT (>40 U/L)	1.323	0.947-1.85	0.101				
AST (>45 U/L)	2.141	1.48-3.096	0.000		1.612	1.065-2.439	0.024
ALB (<35 g/L)	1.839	1.194-2.832	0.006		1.803	1.135-2.862	0.012
TBil (≤20.5 umol/L)	1.033	0.707-1.51	0.865				
HBsAg negative	1.044	0.62-1.757	0.871				
PT (≤13.5 s)	1.096	0.753-1.595	0.632				
ICGR15 (>10%)	1.076	0.738-1.570	0.703				
Child-Pugh score (>6)	1.546	0.981-2.436	0.060				
BCLC stage (B/C/D)	1.544	1.099-2.168	0.012		0.969	0.613-1.533	0.894
Lipiodol dose (>15 ml)	1.848	1.338-2.554	0.000		1.105	0.752-1.624	0.611
Use of gelfoam	1.150	0.763-1.735	0.504				
PD+SD after 1st TACE	2.984	1.945-4.576	0.000		2.608	1.670-4.071	0.000

AFP: alpha fetoprotein; WBC: white blood cell; PLT: blood platelet; ALT: alanine aminotransferase; AST: aspartate aminotransferase; TBIL: total bilirubin; ALB: albumin; HBsAg: hepatitis B surface antigen; PT: prothrombin time; ICG: indocyanine green; BCLC: Barcelona Clinic Liver Cancer; SD: stable disease
